# The Effect of Pregnancy on Urinary Symptoms

**DOI:** 10.7759/cureus.44232

**Published:** 2023-08-28

**Authors:** Ahmet Beyazıt, Ali Ulvi Hakverdi, Kerem Han Gözükara

**Affiliations:** 1 Obstetrics and Gynecology, Mustafa Kemal University, Hatay, TUR; 2 Urology, Adana City Training Hospital, Adana, TUR

**Keywords:** urodynamic, mode of delivery, lower urinary tract symptoms, urinary incontinence, pregnancy

## Abstract

Background

Urinary incontinence is a condition that causes social, medical, or hygienic problems. The increase in the incidence of stress incontinence, particularly with increasing parity, emphasizes the role of pregnancy on the etiology of incontinence and other urinary symptoms. This study aimed to estimate the effect of pregnancy on urinary incontinence and other urinary symptoms with history and urodynamic data.

Methodology

This study was conducted at Mustafa Kemal University, Medical Faculty, Obstetrics and Gynecology Department. A total of 72 pregnant primigravid women without any urinary problems were included in the study. Patients with severe chronic disease, neurological disorders, antepartum hemorrhage, multiple pregnancies, younger than 18, and those with physical and mental disabilities were excluded. All patients were initially evaluated in the first trimester and finally in the sixth week of the postpartum period. Demographic and obstetric data, including urological complaints and urodynamic findings, were recorded.

Results

There were significant increases in nocturia, frequency, dysuria, urgency, and stress urinary incontinence complaints in pregnant women. Urge incontinence was not significantly different after pregnancy. In the postpartum urodynamic studies, nine (12.5%) patients with stress urinary incontinence and six (8.3%) patients with detrusor instability were detected. There was no significant difference between cesarean section and vaginal delivery regarding incontinence.

Conclusions

According to the study findings, pregnant women who were continent before pregnancy could become incontinent after birth according to urodynamic data. However, long-term studies are needed to determine whether this incontinence is temporary. Additionally, according to our results, cesarean section should not be recommended over vaginal delivery only to prevent incontinence.

## Introduction

Female urinary incontinence is a public health problem and affects 25% to 45% of adult women [1]. Urinary incontinence and other urinary symptoms such as frequency and nocturia can impair a woman’s social, professional, and sexual life [2]. Because of these common problems, it is essential to recognize the risk factors for these complaints and counseling patients. In addition, urodynamic studies should be done to establish underlying factors objectively. Urodynamic studies can be invasive (cystometry, pressure-flow study) or noninvasive (uroflowmetry, electromyography). Moreover, urodynamic studies have very few contraindications such as urinary tract infections and being catheterized before urodynamic studies. Although it is a common belief that urodynamic study is not a safe diagnostic tool during pregnancy, it is not correct. There are no harmful effects of urodynamic studies on pregnancy [3]. The development of urinary incontinence is related to pregnancy and birth trauma [3-5]. After the delivery, the prevalence of urinary incontinence in women is high but contradictory results have been reported. Several risk factors for postpartum urinary complaints have been studied, with parity and delivery mode commonly reported as risk factors [5-7]. Both stress and urgency urinary incontinence have been reported to be more common after a vaginal birth than cesarean section. Additionally, other urinary symptoms, especially frequency and nocturia, are common complaints during pregnancy [8,9]. In one study, it was shown that 46% of urinary incontinence complaints started during pregnancy [10]. This study aimed to investigate the effect of pregnancy on urinary incontinence and urinary symptoms using anamnesis and urodynamic examination.

## Materials and methods

The study was conducted in the Obstetrics and Gynecology Department of Mustafa Kemal University Medical Faculty Hospital and was approved by the Ethics Committee of Mustafa Kemal University (protocol number: 13/03/2015/26). Informed consent was obtained from all participants. A total of 72 healthy primigravid pregnant women without any urinary problems were included in the study. Included women did not have any comorbidities such as obesity, asthma, or previous surgeries that would cause incontinence. Patients with severe chronic diseases, neurological disorders, antepartum hemorrhage, multiple pregnancies, younger than 18, and those with physical and mental disabilities were excluded. All patients were initially evaluated in the first trimester and finally in the sixth week of the postpartum period. Demographic and obstetric data, including urological complaints and urodynamic findings, were recorded. At the first and final evaluations, patients were asked questions to investigate urinary symptoms (Table 1). According to the answers, nocturia, frequency, dysuria, urgency, urge incontinence, and stress urinary incontinence complaints were recorded.

**Table 1:** Questions about urinary complaints.

Questions
Do you need to go to the toilet after sleeping at night-time?
Do you urinate more than eight times a day?
Do you experience any discomfort or pain while urinating?
Do you ever have a feeling of instant micturition that you thought would leak urine if you could not reach the toilet?
If the answer was yes to question 4, have you ever missed urine before reaching the toilet?
Do you miss urine when you cough, sneeze, or laugh?

Urodynamic examination

The urodynamic examination was performed twice using the MMS Solar Blue multichannel urodynamics device available at the Obstetrics and Gynecology Clinic. During filling cystometry, isotonic NaCl at room temperature was used at a rate of 30 mL/minute. The first sensation of filling, the first desire to void, a strong desire to void, the maximal cystometric capacity, and pressure lines were recorded. Detrusor contractions that exceeded 15 cmH_2_O during filling that could not be inhibited were noted. Detrusor instability was diagnosed using these findings. During the same procedure, the patient was increasingly made to cough and strain to examine if there was urine leakage from the external meatus. Finally, patients whose bladder was filled with SF were asked to empty the bladder. The postvoid residual urine was then measured using a 16-F Foley catheter.

All data were transferred to the computer, and statistical analyses were done. McNemar test, paired-sample t-test, and chi-square test were performed. P-values <0.05 were considered significant.

## Results

The descriptive and obstetric findings of 72 patients are represented in Table 2. In total, 24 (33.3%) patients delivered by cesarean section, and 48 (66.7%) patients gave vaginal birth. The urinary complaints of the patients who had no urinary symptoms before pregnancy are shown in Table 3. Nocturia, frequency, dysuria, urgency, and stress urinary incontinence complaints increased significantly. Urge incontinence did not show a significant increase compared to pre-pregnancy. Nocturia was observed in 86.1% (62) of the participants during pregnancy. The percentage of participants who urinated eight times or more a day was 77.8% (frequency). Dysuria was detected in 40.3% (29) of patients, and 27.8% (20) of patients experienced urgency. Cystometric values of urodynamic examinations are shown in Table 4. In the first trimester urodynamic examinations, no stress urinary incontinence and detrusor instability were noted. However, in postpartum studies, stress urinary incontinence was found in nine (12.5%) patients (p < 0.05). In our study, stress urinary incontinence was found in three (12.5%) of 24 patients who delivered by cesarean section and in six (12.5%) of 48 patients who had vaginal delivery (p > 0.05). Urodynamic detrusor instability was detected in only one patient who delivered by cesarean section and in five patients who delivered vaginally. There was no statistically significant difference between the two groups (p > 0.05). Detrusor instability was detected in 6 (8.3%) patients (p < 0.05). The mean body mass index (BMI) of the patients was 28.7 kg/m^2^. Based on the BMI of 30 kg/m^2^, which is the limit of obesity, we found seven (16.3%) cases of stress urinary incontinence in 43 patients with BMI <30 kg/m^2^ after pregnancy. In 29 participants with a BMI ≥30 kg/m^2^, stress urinary incontinence was observed in two (6.9%) patients. We found that BMI was not a significant risk factor for stress urinary incontinence.

**Table 2:** Demographic and obstetric findings of patients. BMI: body mass index; SD: standard deviation

Parameters	Minimum	Maximum	Mean ± SD
Age (year)	18	37	25.3 ± 3.8
BMI (kg/m^2^)	24	39	28.7 ± 2.8
The gestational week during delivery (week)	37.6	40.6	38.6 ± 0.7
Birth weight (g)	2,700	4,200	3,346.8 ± 358.3

**Table 3:** Urinary complaints before and during pregnancy. P-values <0.05 are significant. SUI: stress urinary incontinence

Symptoms	Before pregnancy (n = 72)	During pregnancy (n = 72)	P-value
Nocturia	0	62 (86.1%)	<0.05
Frequency	0	56 (77.8%)	<0.05
Dysuria	0	29 (40.3%)	<0.05
Urgency	0	20 (27.8%)	<0.05
SUI	0	13 (18.1%)	<0.05
Urge incontinence	0	4 (5.6%)	>0.05

**Table 4:** Cystometric values. P-values <0.05 are significant. SD: standard deviation

Parameters		Mean	SD	P-value
First sensation (mL)	First trimester	153.7	26.5	<0.05
	Postpartum period	174.5	30.7	
First desire to void (mL)	First trimester	223.7	37.8	<0.05
	Postpartum period	254.2	37.3	
Strong desire to void (mL)	First trimester	320.7	31.2	<0.05
	Postpartum period	376.8	29	
Maximum cystometric capacity (mL)	First trimester	425.8	41.9	<0.05
	Postpartum period	482.4	70.1	
Postvoid residual volume (mL)	First trimester	25.7	23.2	>0.05
	Postpartum period	27.3	23.7	

## Discussion

The increase in the incidence of stress incontinence, particularly with increasing parity, emphasizes the role of pregnancy in the etiology of incontinence. Primigravids are usually young and have stronger connective tissue and pelvic floor muscles than multiparous women in whom pelvic floor muscles and connective tissue are affected by multiple births. Our study included only primigravids to emphasize the effect of pregnancy on urinary symptoms even for the first time.

Many physiological and anatomical changes occur during pregnancy that affect the lower urinary tract. Hormonal factors, changes in the autonomic system, uterine compression, and changes in the pelvic floor muscles are some of the factors. Urinary symptoms, which often appear during pregnancy, cause misconception among women as it is a normal phenomenon after birth. In reality, pregnancy and childbearing increase the risk of urinary incontinence by 2-2.6 times compared to women who have never conceived [11]. The incidence of stress urinary incontinence and urge incontinence were 9.4% and 8.5% in non-childbearing women and 10.4% and 7% in primigravids, respectively, in one study [11]. In our study, we found that lower urinary tract symptoms increased during pregnancy, and nocturia was reported by 86.1% of pregnant women as the most common cause of complaint. Likewise, we found a significant increase in frequency, dysuria, urgency, and stress urinary incontinence symptoms. While urgency was a common complaint, urge incontinence was detected in only four (5.6%) patients in our study group. Anatomical and physiological changes affecting the lower urinary tract during pregnancy and the hormonal environment during pregnancy explain urinary symptoms during pregnancy [12,13]. Detrusor muscles become hypertrophic and hypotonic with increased estrogen and progesterone, increasing bladder capacity [14]. In addition, the bladder anatomically becomes more prone to lower urinary tract symptoms. It settles upwards and anteriorly (more in the abdomen), grows larger, and the trigone becomes more convex [15]. Again, the pressure of the overgrown uterus on the bladder, the increase in glomerular filtration rate, and changes in the urethrovesical angle explain the increase in these symptoms. In one study including 256 pregnant women, voiding symptoms were more common in pregnant women than in the normal population, and nocturia and frequency were the most common symptoms, as reported in our study [16]. In another larger study, Sun et al. found that these symptoms increased during pregnancy, suggesting that the most common symptoms were nocturia (60.1%), stress urinary incontinence (46.1%), and urgency (34.1%) [17]. Frequency was found as 77.8% in our study. Cutner et al. reported frequency as 40% [18]. In the study by Scarpa et al., this rate was 70.3% [19]. Most studies suggest that urge incontinence is not seen in the first trimester of pregnancy. It has been shown that these complaints occur frequently in the second or mostly the third trimester. In our study, urge incontinence was seen in only four (5.6%) patients; however, it was not questioned according to trimesters. These rates were reported as 18% and 8% in two previous studies [14,18].

In our cystometric examination, both first-trimester and postpartum values were compatible with the cystometry parameters in the normal population. The mean first sensation of the filling point was 153 mL in the first trimester and 174 mL in the postpartum period (normal range: 100-250 mL). In our study, we found the mean first desire to void values were 223 mL and 254 mL, respectively (normal range: 200-330 mL). The mean strong desire to void values were 320 mL in the first trimester and 376 mL in the postpartum period (normal range: 350-560 mL). The mean maximal cystometric capacity was 425 mL and 482 mL, respectively (normal range: 300-550 mL) [20]. Additionally, the postvoid residual urine amount was similar to the normal population. In our study, we did not find any significant differences between the first trimester and postpartum residual urine values. In our urodynamic study, we found that nine postpartum women had stress urinary incontinence and six had detrusor instability. This shows that 20.8% of the participants had postpartum incontinence. Meyer et al. Investigated the effect of delivery on incontinence nine weeks after birth and found that 21% had incontinence, similar to our study [21]. The aforementioned findings highlight the effect of the increased uterus and fetal weight on the pelvic floor muscles, pregnancy-related hormonal changes (especially relaxin and progesterone), and birth-related trauma increase the bladder neck and urethral mobility. A study among 500 pregnant women in Brazil [22] revealed a urinary incontinence of 63.8%. However, multiparous pregnant women were included in this study. This shows that the risk of incontinence increases as the number of pregnancies increases. As we included primiparous pregnant women in our study, we could not evaluate parity. Incontinence has been reported less frequently in nulliparous women than in multiparous women. The prevalence of pure stress and mixed incontinence increased with parity, but no significant difference was found in pure urge incontinence [23].

In our study, we found that the mode of delivery had no significant effect on incontinence. However, there are studies advocating the contrary. Many studies suggest that vaginal delivery damages the pelvic facial support, leads to partial denervation of the pelvic floor and urethral muscles, and leads to a reduction in functional urethral length, urethral closure pressure, and maximum urethral pressure [24]. Stress incontinence after cesarean delivery has been reported in 9% of women. This rate supports the theory that full-term pregnancy alone leads to urinary incontinence and that cesarean section has no protective effect [24]. A review by Press et al., similar to our study, suggested that there was no difference in incontinence between cesarean section and vaginal delivery [25]. Long-term studies have also shown no protective effect of cesarean section from incontinence. In the Nordic Trøndelag (EPINCONT) study, 15,307 women were evaluated and compared with women who delivered by cesarean section only and women who had only vaginal delivery. No significant difference was noted between the two groups in the incidence of incontinence in women over the age of 50 [26].

In our study, we found that having a BMI of more than 30 kg/m^2^ during pregnancy did not have a significant effect on incontinence. To our knowledge, there is no randomized controlled study investigating the relationship between incontinence in pregnancy and BMI. However, cohort studies have shown that being overweight before pregnancy increases postpartum incontinence. Burgio et al. suggested that every 5 kg/m^2^ increase in BMI increased risk by 1.2 times [6]. Being overweight has been suggested to increase incontinence by causing increased intra-abdominal pressure, increased intravesical pressure, weakening of the pelvic floor, and increased urethral mobility. The conflicting results of our study originated from the selection of only primiparous pregnant women; therefore, the number of incontinent subjects may be lower.

Our study has some limitations. Inherent to all questionnaire studies is the recall bias and perhaps even the social desirability bias, given the social stigma of urinary incontinence. These biases may have influenced the participants and this fact should be acknowledged. Another weakness of the study is that only the symptoms were questioned but not how it affected the quality of life. We think that if the study had been supported by quality-of-life indicators, it would have been more valuable. The distribution of symptoms according to pregnancy trimesters was not done. We did not differentiate between women who were active and doing exercises, particularly pelvic floor kegel exercises, or who did not exercise. In addition, we do not know the long-term effect of these symptoms. When these individuals are followed for a long time, determining whether the symptoms are permanent or temporary will form the basis of future studies, which will make the studies more valuable.

## Conclusions

Urinary incontinence and other urinary problems are frequently encountered during pregnancy. This situation, which is considered normal by many patients and physicians due to its high frequency, greatly reduces the quality of life of individuals and negatively affects their social and sexual lives. Most pregnant women experience at least one of the urinary symptoms during pregnancy. In our study, it was shown that pregnant women who were continent before pregnancy could become incontinent after birth according to urodynamic data. However, long-term studies are needed to determine whether this incontinence is temporary. There was no significant difference between cesarean section and vaginal delivery regarding incontinence. Cesarean section is not recommended over vaginal delivery only to prevent incontinence.
